# Cancer-Associated Fibroblasts Promote Proliferation of Endometrial Cancer Cells

**DOI:** 10.1371/journal.pone.0068923

**Published:** 2013-07-26

**Authors:** Kavita S. Subramaniam, Seng Tian Tham, Zahurin Mohamed, Yin Ling Woo, Noor Azmi Mat Adenan, Ivy Chung

**Affiliations:** 1 Department of Pharmacology, Faculty of Medicine, University of Malaya, Kuala Lumpur, Malaysia; 2 Department of Obstetrics & Gynecology, Faculty of Medicine, University of Malaya, Kuala Lumpur, Malaysia; 3 Pharmacogenomics Laboratory, Department of Pharmacology, Faculty of Medicine, University of Malaya, Kuala Lumpur, Malaysia; 4 University of Malaya Cancer Research Institute, Faculty of Medicine, University of Malaya, Kuala Lumpur, Malaysia; Robert Wood Johnson Medical School, United States of America

## Abstract

Endometrial cancer is the most commonly diagnosed gynecologic malignancy worldwide; yet the tumor microenvironment, especially the fibroblast cells surrounding the cancer cells, is poorly understood. We established four primary cultures of fibroblasts from human endometrial cancer tissues (cancer-associated fibroblasts, CAFs) using antibody-conjugated magnetic bead isolation. These relatively homogenous fibroblast cultures expressed fibroblast markers (CD90, vimentin and alpha-smooth muscle actin) and hormonal (estrogen and progesterone) receptors. Conditioned media collected from CAFs induced a dose-dependent proliferation of both primary cultures and cell lines of endometrial cancer *in vitro* (175%) when compared to non-treated cells, in contrast to those from normal endometrial fibroblast cell line (51%) (*P*<0.0001). These effects were not observed in fibroblast culture derived from benign endometrial hyperplasia tissues, indicating the specificity of CAFs in affecting endometrial cancer cell proliferation. To determine the mechanism underlying the differential fibroblast effects, we compared the activation of PI3K/Akt and MAPK/Erk pathways in endometrial cancer cells following treatment with normal fibroblasts- and CAFs-conditioned media. Western blot analysis showed that the expression of both phosphorylated forms of Akt and Erk were significantly down-regulated in normal fibroblasts-treated cells, but were up-regulated/maintained in CAFs-treated cells. Treatment with specific inhibitors LY294002 and U0126 reversed the CAFs-mediated cell proliferation (*P*<0.0001), suggesting for a role of these pathways in modulating endometrial cancer cell proliferation. Rapamycin, which targets a downstream molecule in PI3K pathway (mTOR), also suppressed CAFs-induced cell proliferation by inducing apoptosis. Cytokine profiling analysis revealed that CAFs secrete higher levels of macrophage chemoattractant protein (MCP)-1, interleukin (IL)-6, IL-8, RANTES and vascular endothelial growth factor (VEGF) than normal fibroblasts. Our data suggests that in contrast to normal fibroblasts, CAFs may exhibit a pro-tumorigenic effect in the progression of endometrial cancer, and PI3K/Akt and MAPK/Erk signaling may represent critical regulators in how endometrial cancer cells respond to their microenvironment.

## Introduction

Endometrial cancer (EC) is the sixth most commonly diagnosed cancer among women globally, with approximately 288,000 new cases and 50,327 deaths occurring worldwide each year [1]. It is the most common gynecologic malignancy in the United States with an estimate of 47,100 new cases diagnosed in 2012 [2]. Of significance, the incidence and mortality rates for EC have been rising in the developed and developing countries and is expected to rise further with the increasing ageing population and prevalence of obesity [3]. Although the five-year survival for EC is >85%, a subset of endometrial tumors exhibit an aggressive phenotype, characterized by high histological grade, regional lymphovascular invasion and distant metastasis. The prognosis for such tumors is relatively poor, with five-year survival ranging from 16–66% [4].

Approximately 90% of EC cases are sporadic and are classified into type 1 and type 2, according to their etiology and clinical behavior [5]. Type 1 EC represents the majority of sporadic cases, accounting for 70-80% of new cases [5]. Type 1 cancers, mostly endometrioid in histology, are often low-grade tumors with a favorable prognosis. These cancers often present with PTEN, K-*ras* and beta-catenin mutations and increased expression of estrogen receptor [6]. It is suggested that excessive estrogen exposure can lead to atypical endometrial hyperplasia (EH), a benign condition of proliferative endometrial gland [7,8]. Furthermore, atypical EH has been strongly associated with invasive EC in up to 62% endometrial biopsy specimens, suggesting that atypical EH may be the direct precursor to endometrioid type 1 EC [9]. Nevertheless, the primary reason for treatment failure in both type 1 and 2 endometrial cancers is the distant spread of primary tumors (metastasis) [10]. The mechanism leading to this aggressive transformation is yet to be defined. However, studies on other tumor types suggest that surrounding fibroblasts may have important role in tumor progression [11,12].

In the female reproductive tract, fibroblasts can promote epithelial development and differentiation [13,14]. They are responsible for extracellular matrix remodeling and producing paracrine growth factors that control cell proliferation, survival and death [15]. In fact, contribution of cancer-associated fibroblasts (CAFs) in the progression of various cancer types has been studied, for example, in prostate cancer [16–18], pancreatic cancer [12], head and neck cancer [19] and breast cancer [20]. In these tumor models, CAFs enhanced tumor cell proliferation, invasion and chemoresistance. Furthermore, CAFs are also thought to have major roles in modulating tumor angiogenesis, immune cell infiltration and metastatic colonization [21–23]. The involvement of fibroblasts in the progression of EC, however, is relatively under-studied.

Characterization of fibroblast factors in endometrial cancer, while few, are mainly from pathological analyses. Hepatocyte growth factor and cMet expression was significantly correlated with higher stages of EC, although was not prognostic of worse survival [24]. Another study observed that CXCR4 expression was significantly higher in tumors with muscular infiltration, an indicator of metastasis [25]. Interestingly, using primary cultures from endometrial tissues, Arnold et al demonstrated that the secretion from normal endometrial fibroblast cells inhibited the proliferation of Ishikawa cells, a human EC cell line [26]. This observation was further supported by Zhao’s group in which they suggested that such anti-proliferative effect could be due to inhibition of PI3K signaling [27]. Nevertheless, it is still unknown whether CAFs in EC will exhibit an anti-tumor property as with normal endometrial fibroblasts, or a pro-tumor characteristic as with CAFs from other tumor types.

Hence, in this study, we established several primary cultures of human endometrial fibroblast cells from EC tissues, to investigate the effects of CAFs on EC cell proliferation. We further showed that, in contrary to normal endometrial fibroblasts, CAFs promoted EC cell proliferation, in part by modulating PI3K/Akt and MAPK/Erk signaling pathways. We also tested the use of rapamycin, an mTOR inhibitor, as a potential therapeutic agent in inhibiting CAFs-mediated cell proliferation. The study provides new evidence elucidating the pro-tumorigenic role of fibroblasts in the tumorigenesis of EC.

## Materials and Methods

### Chemicals and reagents

U0126 and LY294002 were obtained from Cell Signaling Technology (MA, USA), and rapamycin (sirolimus) was purchased from Clearsynth Labs (Mumbai, India).

### Ethics statement

The study was approved by the Ethical Committee of University Malaya Medical Centre (Ref No. 865.19). Written informed consent was obtained from all participants.

### Human tissues and cell lines

Tissues from four endometrial cancers and one hyperplasia tissue were obtained from women undergoing surgery to remove the tumor part of the endometrium. About 1 g of tissues was transported to the laboratory in media consisting of RPMI1640 (Life Technologies, NY, USA) supplemented with 10% fetal bovine serum (FBS) (Life Technologies, NY, USA) and 1% penicillin/streptomycin (Life Technologies, NY, USA). Tissues were minced to the size of 1 mm^3^ and then digested with 2 mg/ml of collagenase II for EC tissues and with collagenase I for hyperplasia tissue (Worthington, New Jersey, USA) in a rotator for 1 hour at 37 ^°^C. Post digestion, tissues were washed and cultured in RPMI1640 media supplemented with 10% FBS and 1% penicillin/streptomycin at 37 ^°^C. Cultures were maintained by media change every 72 hours and sub-cultured after reaching confluency. Human endometrial cancer cell lines, ECC-1 (CRL-2923) and HEC-1-A (HTB 112) and immortalized human normal endometrial fibroblast cell line, T-HESC (CRL-4003) were purchased from American Type Culture Collection (Bethesda, MD, USA) and were cultured in media according to manufacturer’s protocol.

### Isolation of primary epithelial and stromal cells

All cultured primary cells obtained from surgical tissues were subjected to stromal cell isolation using anti-fibroblast magnetic microbeads (Miltenyi Biotech, Cologne, Germany). Briefly, 1x10^6^ cells were centrifuged at 300x*g* for 10 minutes. Cell pellets were then resuspended in 100 µl of buffer containing a final concentration of 0.5% bovine serum albumin and 2 mM ethylenediaminetetraacetic acid dissolved in pH 7.2, calcium and magnesium free phosphate-buffered saline and incubated with 20 µl of human anti-fibroblast microbeads antibody (Miltenyi Biotec, CA, USA) for 1 hour. Cells were then separated using MiniMACS™ cell separator (Miltenyi Biotec, CA, USA). Isolated cells were then continued to be cultured in the media mentioned above. Epithelial cells population was also harvested using similar method, using human CD326 (EpCAM) magnetic microbeads antibody (Miltenyi Biotec, CA, USA).

### Flow cytometry analysis

Cultured cells were trypsinized and 1x10^6^ single cell suspension was blocked with 10% normal goat serum (Biowest, Nuaillé, France) before staining with AlexaFluor 647 (AF647)-conjugated human epithelial cell adhesion molecule (EpCAM) and PE-conjugated human CD90 antibodies (Biolegend, San Diego, USA). Isotype controls used were AF647 mouse IgG2b,κ and PE mouse IgG1, κ, respectively. Staining was then analyzed using BD FACSCanto II flow cytometer and the results were viewed using FACS DiVa software (BD Bioscience, California, USA).

### Quantitative real time polymerase chain reaction (qRT-PCR)

Total RNA were extracted from cultured cells using TRIzol (Invitrogen, California, USA) and 1 µg RNA was converted into cDNA using DyNAmo cDNA synthesis kit (Finnzymes, Vantaa, Finland). Sequence for primers used to detect epithelial cell markers (EpCAM, E-cadherin, cytokeratin 8) and fibroblast cell markers (alpha-smooth muscle actin (α-SMA) and vimentin) are listed in Table 1. qRT-PCR was performed using ABI StepOne Plus (Applied Biosystem, California, USA) in 35 cycles using 5x HOT FIREPol EvaGreen qPCR Mix (Solis Biodyne, Tartu, Estonia), 10 pmol/µl forward and reverse primer, 10 ng/µl cDNA template and PCR grade H _2_O. Assays were performed at least in triplicate, and the mean values were used to calculate relative expression levels using the ∆∆C(t) method. Expression levels were first normalized to housekeeping GAPDH gene. Next, gene expression tested in epithelial cells was compared to the corresponding mRNA level observed in a representative fibroblast cells (T-HESC), and similarly, those genes reported expressed in fibroblast cells were compared with their corresponding mRNA level in a representative epithelial cells (ECC-1).

**Table 1:** *Primerssequences* used for qRT-PCR.

Gene	Primers		Source
**EpCAM**	Forward	5’-AATGTGTGTGCGTGGGA-3’	[79]
	Reverse	5’-TTCAAGATTGGTAAAGCCAGT-3’	
**E-cadherin**	Forward	5’-TTTGTACAGATGGGGTCTTGC-3’	[80]
	Reverse	5’-CAAGCCCACTTTTCATAGTTCC-3’	
**Cytokeratin 8**	Forward	5’-CTGGTGGAGGACTTCAAGAAC-3’	[81]
	Reverse	5’-GACCTCAGCAATGATGCTGTC-3’	
**αSMA**	Forward	5’-GACGAAGCACAGAGCAAAAGAG-3’	[82]
	Reverse	5’-TGGTGATGATGCCATGTTCTATCG-3’	
**Vimentin**	Forward	5’-TGGCACGTCTTGACCTTGAA-3’	[83]
	Reverse	5’-GGTCATCGTGATGCTGAGAA-3’	

### Preparation of fibroblast cells-conditioned media

Fibroblast cells were seeded and cultured in complete media for 24 hours, before being cultured in media containing 2% FBS for the following 72 hours. Conditioned medium was collected using Amicon ultra centrifugal filters (Merck Milipore, Massachusetts, USA) by centrifugation at 5000x*g* at 4 ^°^C for 1 hour. Protein in the concentrated media was quantified using Bradford assay (Biorad, CA, USA).

### Methyl thiazolyl tetrazolium (MTT) assay

Proliferation of epithelial cells was assessed by methyl thiazolyl tetrazolium (MTT) test. Briefly, cells were seeded in complete media at 1-3 x10^3^ cells/well in 96-well plates. At 24 hours post seeding, the cells were treated with either complete media, media with 2% FBS, fibroblast-conditioned media and/or inhibitors for 72 hours. At the end of treatment, 20 µl of MTT solution (5 mg/ml) was added to each well. Following 4 hours incubation at 37 ^°^C, 100 µl of 10% sodium dodecyl sulfate were added to dissolve the formazan crystals by additional 4 hours incubation at 37 ^°^C. Absorbance was measured using spectrometer at 575 nm with reference of 650 nm.

### Total protein extraction and western blotting

ECC-1 cells were seeded at 1x10^4^ cells/well in 6-well plates in complete media. At 24 hours post seeding, the cells were treated with either complete media, media with 2% FBS, fibroblasts-conditioned media and/or inhibitors for 72 hours. Protein lysates were collected by scraping the cells in cold lysis buffer containing final concentration of 0.1% Triton-X, 0.1% SDS, 50 mM Tris, 150 mM NaCl, 1x phosphatase and 1x protease inhibitors. Protein concentration was quantified using Bradford assay (Biorad, CA, USA). Approximately 20 µg of protein were resolved on 10% sodium dodecyl sulfate polyacrylamide gel electrophoresis before being transferred onto polyvinylidene difluoride membrane. Antibodies used were rabbit anti-human Akt, phospho-Akt, Erk, phospho-Erk and β-actin (Cell Signaling Technology, USA). Blots were visualized using ECL prime western blotting detection reagent (Amersham, GE Healthcare Lifesciences, Sweden) using gel documentation system (Biospectrum 410, UVP) and Vision Works LS software (CA, USA)

### Enzyme-linked immunosorbent assay (ELISA)

The levels of phosphorylated-Akt and phosphorylated-Erk in ECC-1 cells treated with 1 µg/µl conditioned media from T-HESC and CAFs were quantified using ELISA kits (Cell Signaling Technology, Danvers, MA, USA). Briefly, 96-well plates were coated with diluted capture antibody overnight, before blocking and incubation with cell lysates for 2 hours each. Diluted detection antibody was incubated for 1 hour prior to incubation of secondary antibody for another 30 minutes. TMB substrate was then added to each well for 15 minutes for color development before terminated with STOP solution. Absorbance was read at 450nm wavelength using spectrometer (Tecan Systems, CA, USA). Data shown were average of at least three replicates normalized with readings from ECC-1 cells treated with media containing 2% FBS.

### Identification and measurement of cytokine levels

Cytokines secreted by normal fibroblasts (T-HESC) and cancer-associated fibroblasts (CAFs of EC6, 7 and 11) were measured using Raybiotech Quantibody Human Cytokine Array (Raybiotech, Georgia, USA) according to the manufacturer’s protocol. Briefly, conditioned media was prepared from 72 hours-cultured fibroblasts as described above. 100 µg protein from each fibroblast secretion was added into respective array well and incubated overnight at 4°C. Each well was washed, incubated with reconstituted antibody cocktail for 2 hours at room temperature, washed again, before addition of Cy3-conjugated streptavidin. Following additional extensive washing, fluorescence signal was measured using the Agilent High Resolution Microarray Scanner (C-model), and raw signal data were extracted from TIFF image with GenePixPro 6.1 before analyzed with Q-Analyzer. Cytokine levels from each fibroblast secretion were compared with media containing 2% FBS (control). Data shown for each sample were average of fluorescence intensity from four array wells.

### Statistical analysis

Statistical analysis that assessed the differences between means of control and test group was performed using Student’s *t*-test on IBM SPSS Statistics 20. A *P*-value <0.05 was considered to be statistically significant.

## Results

### Isolation of cancer-associated fibroblast cells from human endometrial cancer tissues

**Figure 1:**
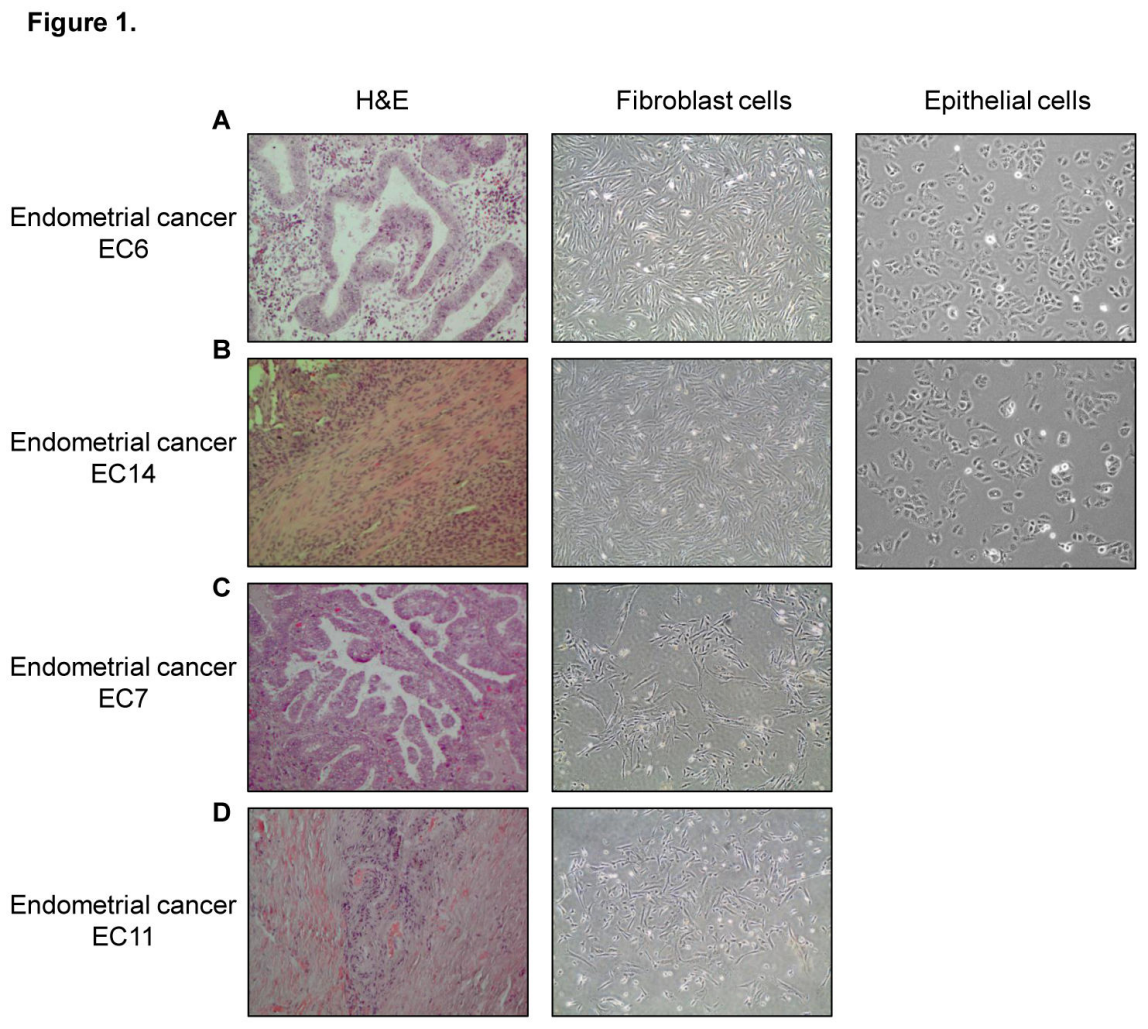
Establishment of epithelial and fibroblast cells from human endometrial cancer tissues. Hematoxylin & eosin (H and E) staining of tissues and phase contrast images of established primary cells isolated from endometrial cancer: EC6 (**A**), EC14 (**B**), EC7 (**C**) and EC11 (**D**). Magnification: 100x. Subsequently these were digested with collagenase and cultured, prior to epithelial and fibroblast cell isolation using CD326 (EpCAM) and anti-fibroblast labeled magnetic beads.

To establish primary fibroblast cells from endometrial tissues, human endometrial cancer (EC) tissues (EC6, 7, 11 and 14) were digested with collagenase, followed by cell isolation using magnetic beads conjugated with anti-fibroblast antibody. For EC6 and EC14, negatively selected cells were then subjected to anti-CD326 conjugated magnetic beads for enrichment of the epithelial counterpart. The isolated epithelial and fibroblast cells were designated as ‘Ep’ and ‘Fib’, respectively. As shown in Figure **1**, there was a clear difference in morphology between epithelial cells (EC6-Ep and EC14-Ep) and fibroblast cells (EC6-Fib, EC7-Fib, EC11-Fib, EC14-Fib). Epithelial cells exhibited rose petal-shaped morphology and tend to grow in colonies, while the stromal cells displayed elongated spindle-shaped features.

**Figure 2:**
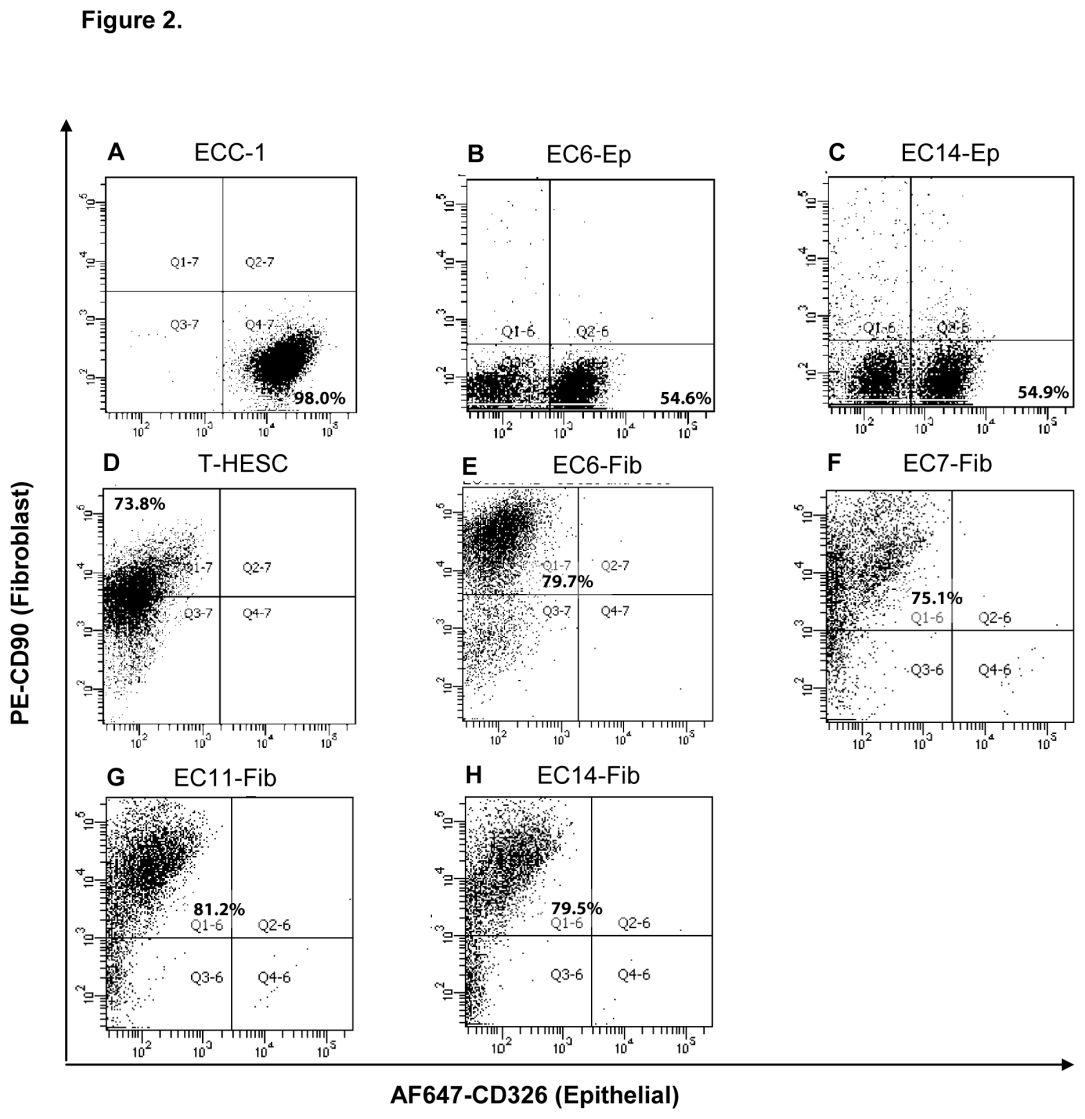
EpCAM and CD90 expression in established primary culture. Flow cytometry analysis of primary epithelial and fibroblast cells isolated from endometrial cancer tissues was performed after labeling cells with CD326 antibody-conjugated with AlexaFluor647 and CD90 antibody-conjugated with PE. Epithelial endometrial cell line, ECC-1 (**A**), primary epithelial cells EC6-Ep (**B**) and EC14-Ep (**C**), normal endometrial stromal cell line, T-HESC (**D**) and primary fibroblast cells, EC6-Fib (**E**), EC7-Fib (**F**), EC11-Fib (**G**) and EC14-Fib (**H**).

To determine the purity of the isolated epithelial and fibroblast cell cultures, we stained the cells with both epithelial marker, Alexa Fluor 647-conjugated EpCAM and fibroblast marker, PE-conjugated CD90 antibodies. Human endometrial adenocarcinoma cancer cell line, ECC-1 showed high expression of EpCAM (98%) whereas, human normal endometrial fibroblast cell line, T-HESC demonstrated high expression of CD90 (74%) (Figure 2A, D). Staining with isotype antibody controls showed minimal binding, indicating specificity of the primary antibodies (data not shown). Epithelial cells isolated from EC6 and 14 (EC6-Ep and EC14-Ep) showed moderate expression of EpCAM (55%) with no evidence of CD90 expression, indicating that this epithelial culture was not contaminated with fibroblast cells (Figure 2B, C). In contrast, the fibroblast cells isolated from EC tissues (EC6-Fib, EC7-Fib, EC11-Fib, and EC14-Fib) were negative for EpCAM expression but highly positive for the fibroblast marker CD90 (75-81%), indicating that the isolated fibroblast cells were relatively pure and free of epithelial cell contamination (Figure 2E–H). All the primary cells used were below passage 10 post culture, to maintain the closest phenotype to the primary tissues.

### Molecular characterization of endometrial primary cultures

**Figure 3:**
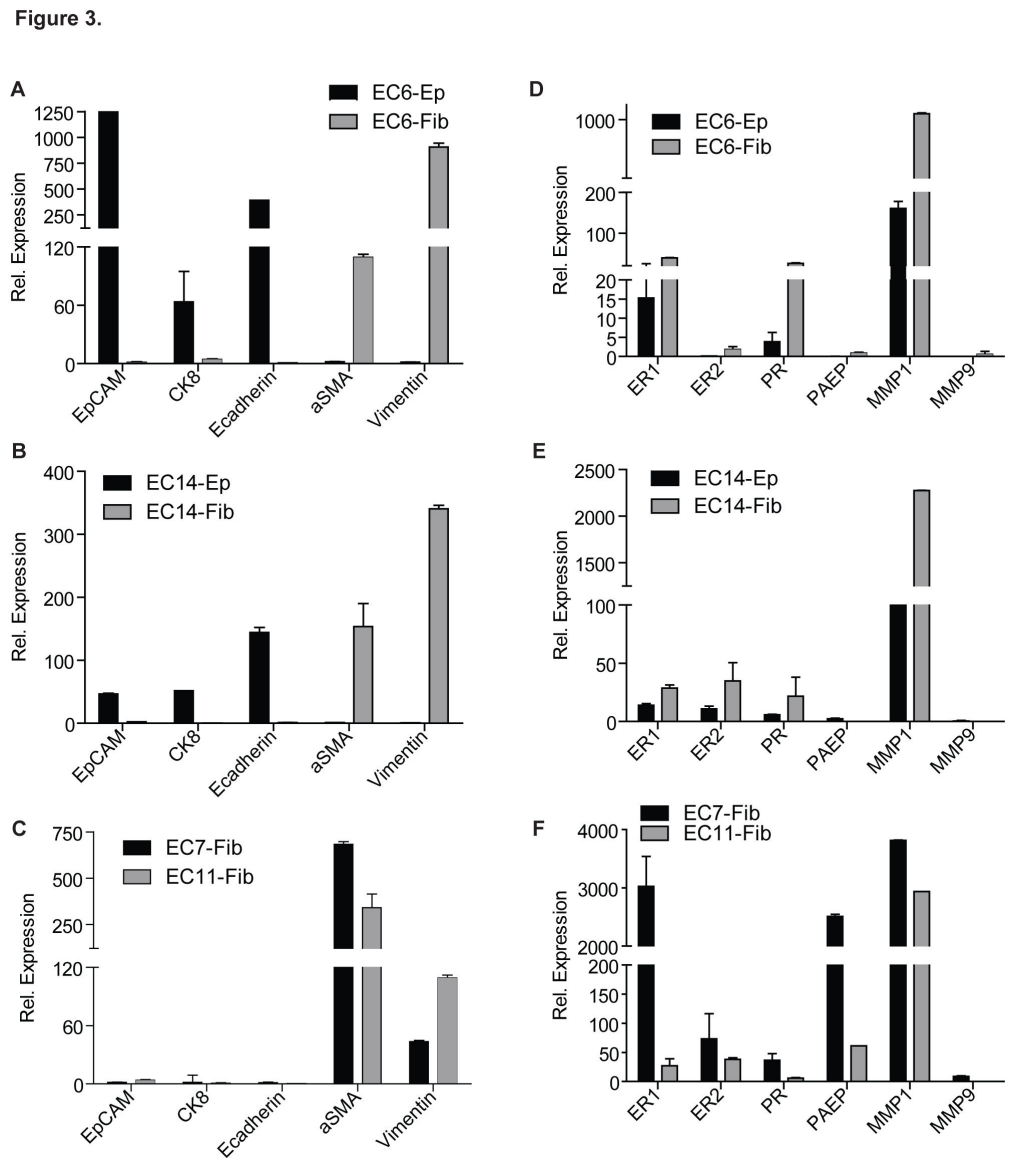
Expression of epithelial, fibroblast and endometrial markers in the primary epithelial and fibroblast cell cultures. Total RNA was subjected to quantitative real-time PCR analysis of epithelial markers (EpCAM, cytokeratin 8, E-cadherin), fibroblast markers (alpha-smooth muscle actin (αSMA), vimentin) (**A**–**C**), estrogen receptor 1 and 2 (ER1, 2), progesterone receptor (PR), progestagen-associated endometrial protein (PAEP) and matrix metalloproteinase 1 and 9 (MMP1, 9) (**D**–**F**). Data, average; error bars, S.E.M. Data shown are representative of three independent experiments.

To further characterize the isolated epithelial and fibroblast cells, we performed quantitative RT-PCR to determine the expression of several epithelial and fibroblast markers. Epithelial EC6-Ep and EC14-Ep cells showed high expression of EpCAM, cytokeratin 8 and E-cadherin, with low expression of vimentin and α-SMA (Figure 3A, B). The expression level shown was normalized with the level of GAPDH. In contrast, the four fibroblast cells isolated from endometrial cancer tissues (EC6-Fib, EC7-Fib, EC11-Fib and EC14-Fib) showed greater expression of vimentin and α-SMA, with low expression of EpCAM, E-cadherin and cytokeratin 8 (Figure 3A–C). These data suggested that we were successful in isolating relatively pure epithelial cells with their fibroblast counterparts from the endometrial cancer tissues.

In addition, we also determined that both epithelial and fibroblast cells from EC tissues expressed varying degrees of estrogen and progesterone receptors (ER1, ER2 and PR) (Figure 3D–F), consistent with the observation that EC are hormone-responsive tumors. We measured the mRNA expression of three commonly secreted proteins by the endometrium, progestagen-associated endometrial protein (PAEP) and matrix metalloproteinase 1 and 9 (MMP1, 9) in these cells. As shown in Figure **3D–F**, PAEP were mainly expressed by fibroblasts, and greater MMP1 expression was observed compared to that of MMP9 in both epithelial and fibroblast cells. Taken together, our data strongly suggested that these primary epithelial and fibroblast cells were maintaining their *in vivo* phenotypes.

### Differential effects of endometrial fibroblast secretion on endometrial cancer cells

**Figure 4:**
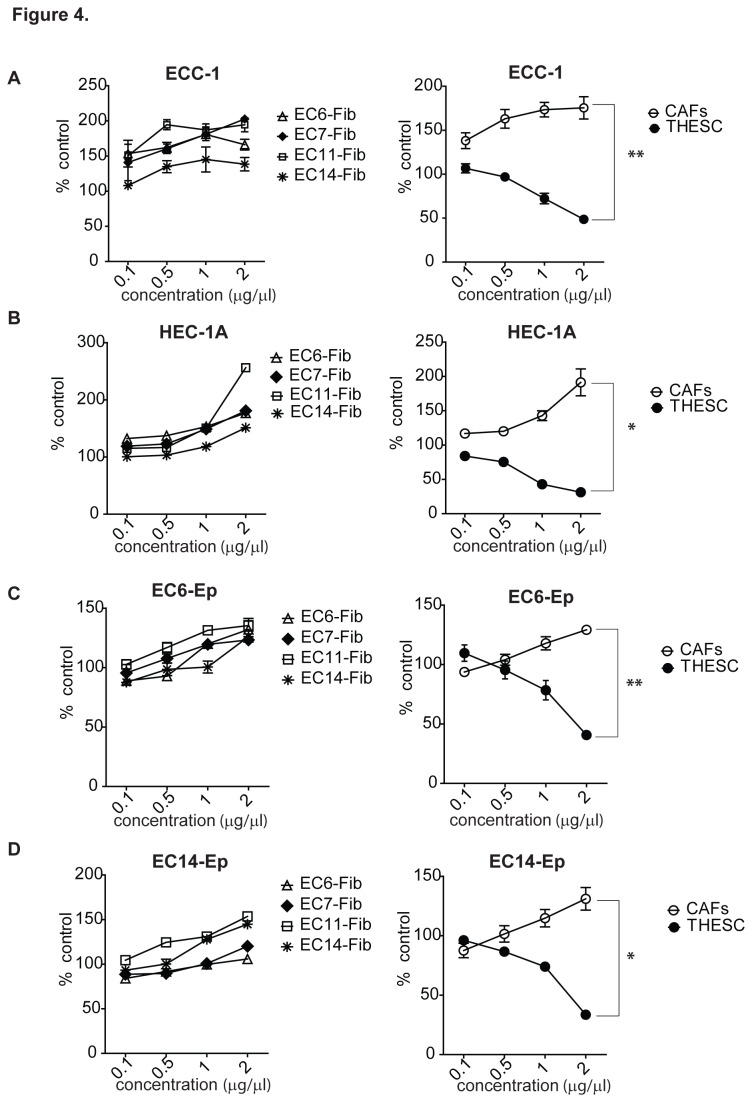
Differential effects of normal and cancer-associated endometrial fibroblast on EC cell proliferation. ECC-1 (**A**) and HEC-1A (**B**) cell lines and EC6-Ep (**C**) and EC14-Ep (**D**) primary endometrial cancer cells were tested with conditioned media prepared from cancer-associated fibroblasts (EC6-Fib, EC7-Fib, EC11-Fib and EC14-Fib) and normal endometrial fibroblast cell line (T-HESC) for 72 hours. Cell viability was examined using MTT assay and were normalized with control (media containing 2% FBS). CAFs shown are the average of all the four cancer-associated fibroblasts tested. Data, average; error bars, S.E.M. *, *P*<0.005;. **, *P*<0.0001. Data shown are representative of three independent experiments.

It had been previously shown that the secretions from normal endometrial fibroblast cells were growth inhibitory to the endometrial cancer cell line, Ishikawa cells [26]. Consistently, conditioned media from normal endometrial fibroblast T-HESC cell line inhibited the proliferation of ECC-1 and HEC-1A, in a dose-dependent manner (Figure 4A, B). At 2 µg/µl, we observed a significant 51% and 69% growth inhibition in ECC-1 and HEC-1A, respectively. Similarly, primary endometrial cancer cells, EC6-Ep and EC14-Ep were also growth inhibited by T-HESC conditioned media (EC6-Ep: 60%; EC14-Ep: 67%) (Figure 4C, D).

To determine and compare the effects of CAFs secretions on endometrial cancer cells, we harvested conditioned media from 72 hours-cultured fibroblast cells, and then treated ECC-1 and HEC-1A human endometrial cancer cell lines for 72 hours. Interestingly, conditioned media from cancer-associated fibroblasts (EC6-Fib, EC7-Fib, EC11-Fib and EC14-Fib) induced a contrasting effect: the growths of both the primary endometrial cancer cells (EC6-Ep and EC14-Ep) and the commercial endometrial cancer cells (ECC-1 and HEC-1A) were markedly enhanced in a dose-dependent manner (Figure 4A–D). Greater effects were seen with ECC-1 and HEC-1A cell lines than in primary cultures, EC6-Ep and EC14-Ep. Among the CAFs, EC-11-Fib demonstrated the most growth-promoting effects, ranging 135% to 274% growth when compared to untreated cells. When these individual CAF effects were combined (labeled as CAFs), there was a significant difference of percent cell growth mediated by CAFs and T-HESC at 2 µg/µl treatment (*P*<0.05) (Figure 4A–D).

**Figure 5:**
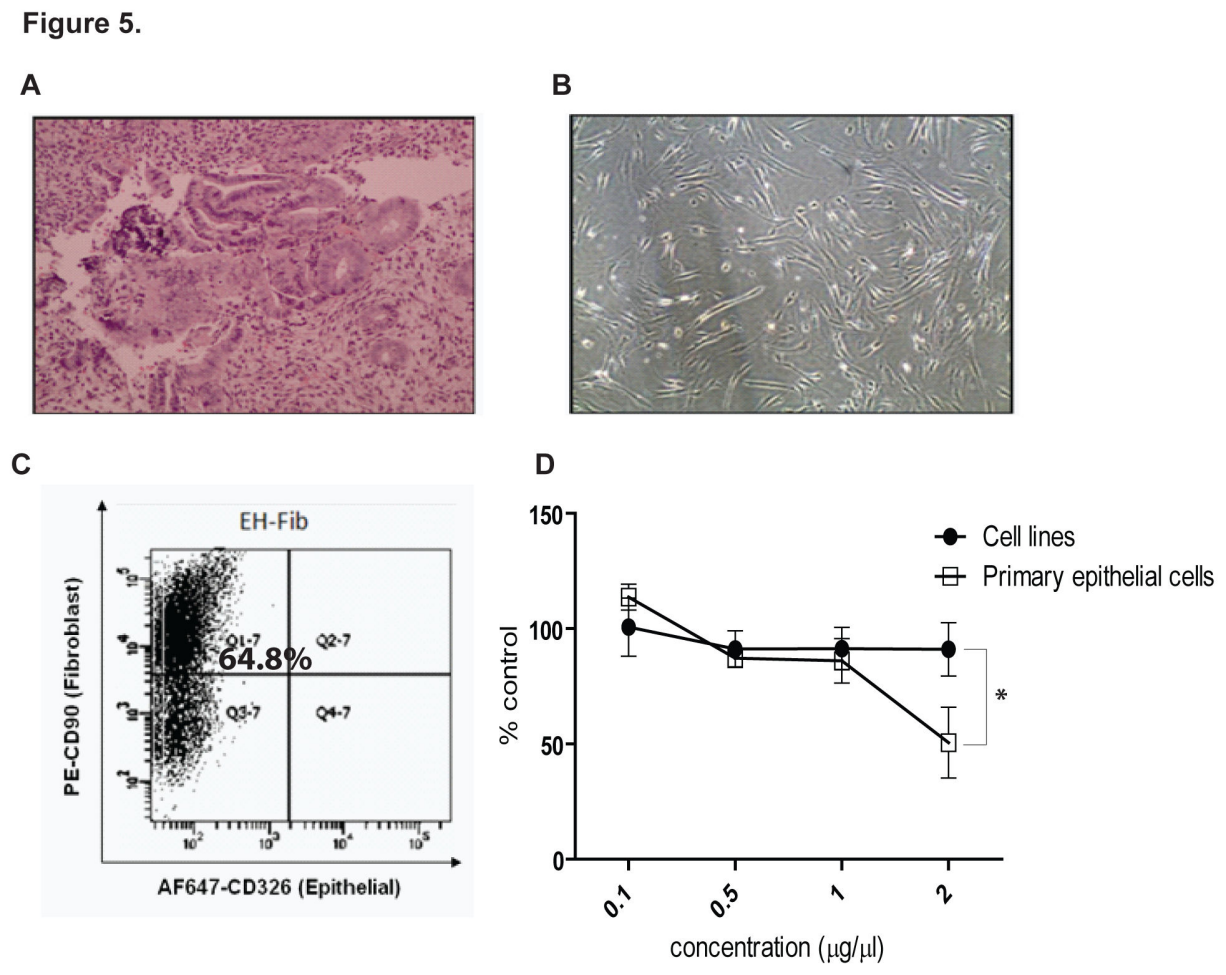
Fibroblast isolated from endometrial hyperplasia tissue did not promote endometrial cancer cell proliferation. Hematoxylin and eosin staining (**A**) and phase contrast image (**B**) of fibroblast cells isolated from human atypical hyperplasia tissue (EH-Fib); Magnification, 100x. (**C**) Flow cytometry analysis of EH-Fib stained with epithelial marker CD326-Alexa Fluor 647 and fibroblast marker CD90-PE. (**D**) Cell viability assay determining the effects of EH-Fib conditioned media on endometrial cancer cell lines (ECC-1 and HEC-1A) and primary epithelial cells (EC6-Ep and EC14-Ep) after 72 hours treatment. Data, average; error bars, S.E.M. *, *P*<0.01. Data shown are representative of three independent experiments.

To exclude the possibility that the CAFs-growth promoting effects were due to our cell culture procedures, we isolated fibroblasts from an atypical hyperplasia tissue, a benign endometrium condition, using similar approach. The isolated fibroblasts (EH-Fib) showed similar fibroblastic morphology *in vitro*, and expressed high level of CD90 (65%) (Figure 5A–C). Using the conditioned media from these cells, we examined their effects on cell proliferation of both the cancer cell lines (ECC-1 and HEC-1A) and primary epithelial cells (EC6-Ep and EC14-Ep). As shown in Figure **5D**, EH-Fib conditioned media did not significantly affect the proliferation of ECC-1 and HEC-1A cells. However, when tested on primary epithelial cells EC6-Ep and EC14-Ep, EH-Fib inhibited growth in a dose-dependent manner, with an average of 69% at 2 µg/µl concentration (Figure 5D). This data suggests that the growth-promoting effects by CAFs is specific, and not due to selection by our experimental procedure.

### Activation of PI3K/Akt and MAPK/Erk pathways in cancer-associated fibroblast-mediated endometrial cancer cell proliferation

**Figure 6:**
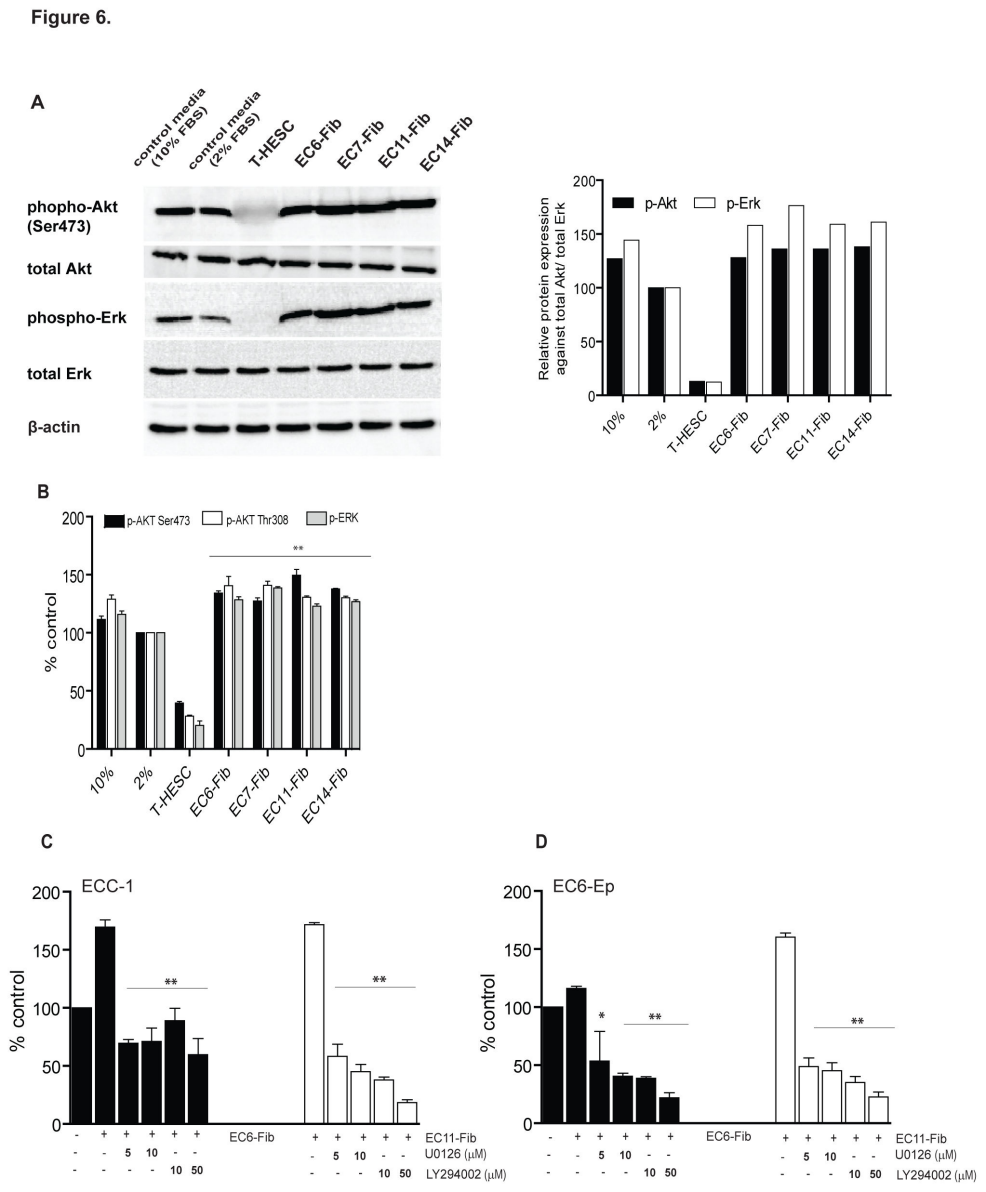
Role of PI3K/Akt and MAPK/Erk signaling pathways in cancer-associated fibroblast-mediated endometrial cell proliferation. (**A**) Western blot analysis of phosphorylated-Akt (Ser473) and -Erk protein expression in ECC-1 cells after treated with either normal endometrial fibroblast T-HESC cells or cancer-associated fibroblast cells (EC6-Fib, EC7-Fib, EC11-Fib and EC14-Fib). Densitometry analysis compared the relative expression level of p-Akt and p-Erk to their total protein level. (**B**) Quantitative analysis of phosphorylated-Akt (Ser473 and Thr308) and phosphorylated-Erk levels in ECC-1 cells after treated with either T-HESC or CAFs, in comparison to cells treated with control (media containing 2% FBS). ECC-1 (**C**) and EC6-Ep (**D**) cells were treated with either PI3K pathway selective inhibitor (LY294002) or Erk pathway selective inhibitor (U0126) in the presence of cancer-associated fibroblasts conditioned media (1 µg/µl) for 72 hours. Data shown are cell viability after normalized with control (media containing 2% FBS). Data, average; error bars, S.E.M. *, *P*<0.05; **, *P*<0.0001. Data shown are representative of two independent experiments.

To elucidate the mechanism underlying the growth promoting effects of CAFs secretion on EC, we determined the activation of PI3K/Akt and MAPK/Erk, two major survival pathways implicated in endometrial cancer. Consistent with previous study [27], treatment of normal fibroblast T-HESC-conditioned media markedly reduced phospho-Akt and phospho-Erk protein expression in ECC-1 cells, as shown with Western blot and ELISA assays (Figure 6A, B). In contrast, phospho-Akt protein level was moderately elevated when ECC-1 cells were treated with EC6-Fib, EC7-Fib, EC11-Fib and EC14-Fib (Figure 6A, B). In addition, CAFs-treated ECC-1 cells also demonstrated increased the level of phospho-Erk, when compared to those treated with control media (Figure 6A, B).

To further investigate the functional role of PI3K/Akt and MAPK/Erk pathways in CAFs-mediated cell proliferation, we next treated ECC-1 and EC6-Ep cells with PI3K selective inhibitor (LY294002) and Erk selective inhibitor (U0126) in the presence of EC6-Fib and EC11-Fib conditioned media for 72 hours. Both LY294002 and U0126 significantly reduced CAFs-mediated cell proliferation in these cells (*P*<0.0001) (Figure 6C, D). Notably, U0126 caused a greater growth inhibitory effect in EC cells treated with EC11-Fib conditioned media. The effects of LY294002 and U0126 in inhibiting endometrial cancer cell proliferation was only evident in the presence of CAFs secretion media, as these inhibitors minimally affected cell proliferation in control media (Figure **S1**). These inhibitors also exerted similar effects on other EC cells, HEC-1A and EC14-Ep (Figure **S1**). These data suggest that activation status of PI3K/Akt and/or MAPK/Erk pathways may be the key point by which fibroblasts from both normal and cancer conditions regulate endometrial cancer cell proliferation.

**Figure 7:**
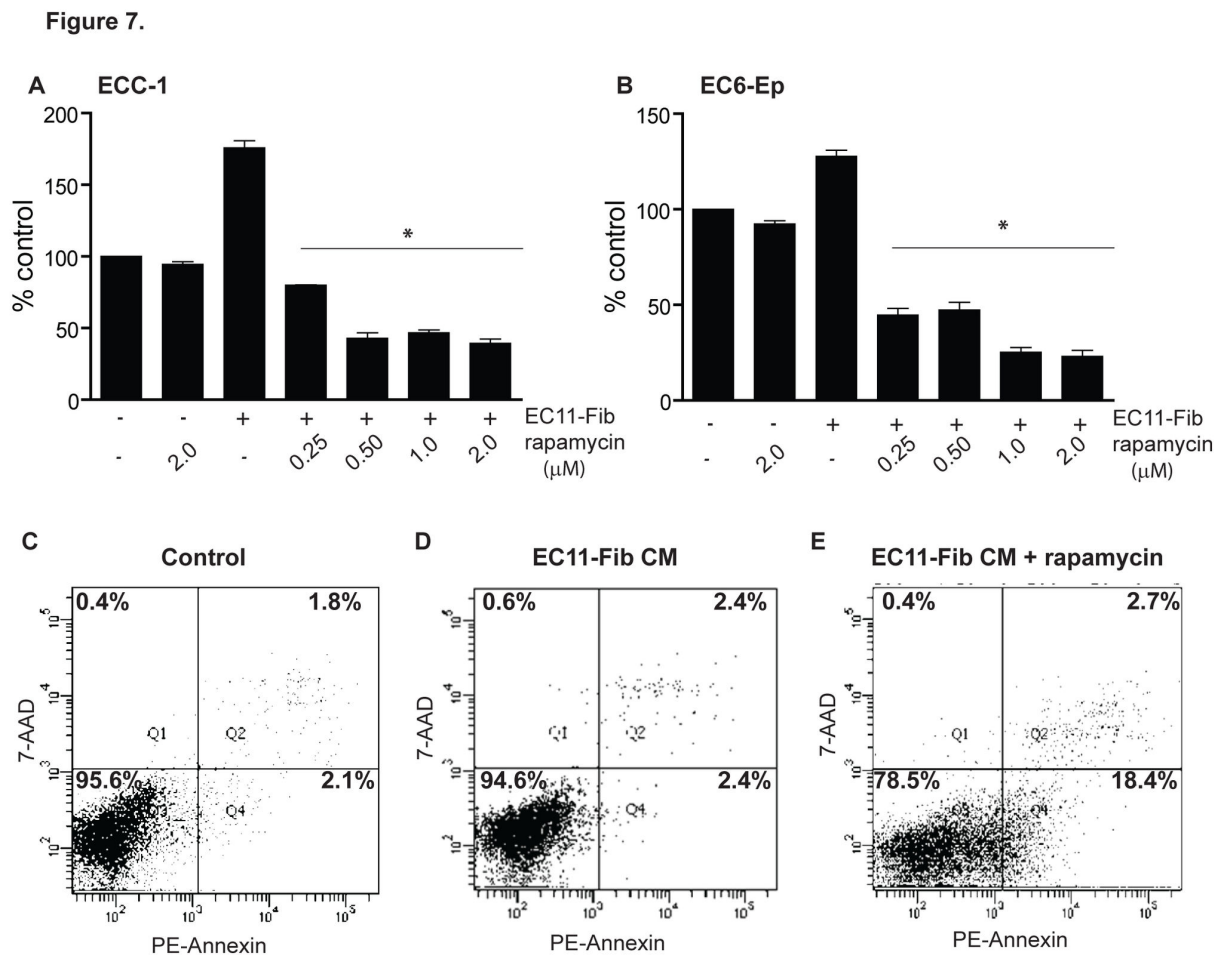
Mechanism of action of rapamycin as PI3K/mTOR pathway inhibitor. ECC-1 cell line (**A**) and EC6-Ep primary epithelial cell (**B**) were treated with increasing dose of rapamycin for 72 hours under the influence of control media (media containing 2% FBS) or 1 µg/µl EC11-Fib conditioned media. (**C**–**E**) ECC-1 cells treated with 2 µM of rapamycin with or without 1 µg/µl EC11-Fib conditioned media for 72 hours, were stained with annexin V-PE and 7-AAD before analyzed with flow cytometry. Data, average; error bars, S.E.M. *, *P*<0.0001 compared to EC11-Fib treated cells. Data shown are representative of two independent experiments.

We further evaluated whether rapamycin, a known PI3K downstream inhibitor, can be clinically useful in reversing CAFs-mediated EC cell proliferation. In the presence of EC11-Fib conditioned media, treatment of rapamycin for 72 hours effectively inhibited ECC-1 and EC6-Ep cell proliferation (*P*<0.0001) (Fig. 7A, B). At the highest dose tested (2 µM), rapamycin reduced ECC-1 cells from 180% (conditioned media treatment alone) to 40% (conditioned media and rapamycin treatment) (*P*<0.0001), while minimal inhibition was observed when cells were cultured in control media (Fig.7A, B). Similar result was observed with the effects of rapamycin on other EC cells, HEC-1A and EC14-Ep (Figure **S2**). Using annexin V labeling, we further determined that rapamycin inhibited CAFs-mediated EC cell proliferation *via* induction of apoptosis (Figure 7C–E). Treatment of ECC-1 with 1 µg/µl EC11-Fib conditioned media for 72 hours did not significantly affect the percentage of apoptotic cells; however, concurrent treatment with 2 µM rapamycin resulted in an increase of apoptotic cell population (annexin V-positive cells) from 4.8% to 21.1%. This suggests that rapamycin and its analogs may be useful in limiting CAFs-mediated EC cell proliferation in the clinical setting.

### Profiling of cytokines secreted by normal and cancer-associated endometrial fibroblasts

**Figure 8:**
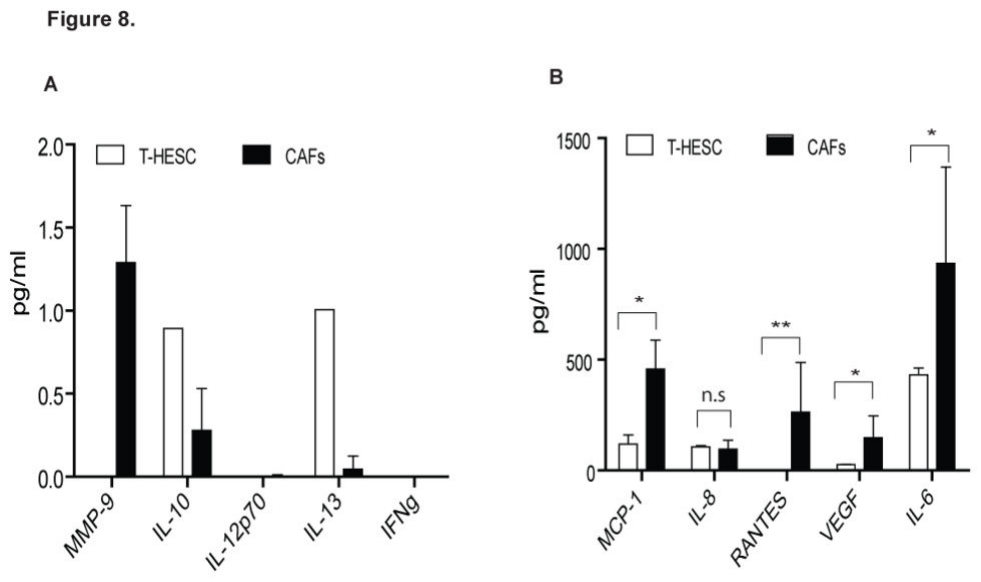
Identification and measurement of cytokines secreted by normal and cancer-associated fibroblasts. Conditioned media from T-HESC (normal fibroblast) and CAFs of EC-6, 7 and 11 (collectively as CAFs) were subjected to antibody array measuring the levels of 10 different cytokines. (**A**) Matrix metalloproteinase-9 (MMP-9), interleukin (IL)-10, IL-12p70, IL-13 and interferon gamma (IFNg) were minimally secreted by both T-HESC and CAFs. No significant difference was found between T-HESC and CAFs due to low detection levels. (**B**) CAFs secreted greater amount of macrophage chemoattractant protein-1 (MCP-1), IL-8, IL-6, RANTES and vascular endothelial growth factor (VEGF) when compared to T-HESC. Data shown were average of fluorescence intensity from four different array wells; error bars, S.E.M. *, *P*<0.05, **, *P*<0.005.

To determine the secretory factors responsible for CAFs-mediated cell proliferation, we performed an antibody array comparing levels of different cytokines in the conditioned media harvested from CAFs and normal fibroblasts. There was a minute amount (less than 2 pg/ml) of interleukin (IL)-10, IL-12p70, IL-13 and matrix metalloproteinase-9 (MMP-9) found in the secretion from both normal fibroblast T-HESC and CAFs (Figure 8A). Interferon gamma (IFNg) was not determined in any fibroblast secretions (Figure 8A). Interestingly, several cytokines including IL-6, IL-8, macrophage chemoattractant protein-1 (MCP-1), chemokine (C-C motif) ligand 5 (CCL5 or RANTES) and vascular endothelial growth factor (VEGF) were found highly expressed by these fibroblasts. There was no significant difference between the levels of IL-8 secreted by T-HESC and CAFs (*P*>0.75) (Figure 8B). However, a significant higher levels of IL-6 (~2-fold), MCP-1 (~4-fold), VEGF (~5-fold) and RANTES (~260-fold) levels were secreted by CAFs when compared to those by T-HESC (*P*<0.05) (Figure 8B). The levels for each cytokine in individual fibroblast secretion are shown in Figure **S3**.

## Discussion

While cancer-associated fibroblasts (CAFs) have been implicated in the progression of many cancer types [12,18], their role in EC have not been defined. It has not been previously described whether CAFs in EC exhibit pro-malignant characteristics or anti-malignant properties. To study this, a relatively pure cancer-associated fibroblast (CAFs) cell population was established from human endometrial cancer tissues and compared to normal fibroblasts. In contrast to the effects of normal fibroblasts, these CAFs exerted growth-promoting effects on endometrial cancer cells. The analysis revealed that PI3K/Akt and MAPK/Erk may be the common key pathways by which both normal and cancer fibroblasts regulate cancer cell proliferation. High secretion of one or more cytokines by CAFs (IL-6, IL-8, VEGF, RANTES and MCP-1) may potentially mediate the activation of these pathways to induce EC cell proliferation. This study provides evidence to support the notion that the underlying fibroblasts may directly influence the progression of endometrial cancer.

Stromal cells are key players in directing growth and differentiation of the overlaying epithelium in the endometrium [28,29]. While in most studies stromal cells were isolated using various filtration methods [26,28,30], we adopted a magnetic-based cell sorting method to obtain relatively pure fibroblast cultures from human endometrial cancer tissues [31–34]. Consistent with previous studies, the resultant fibroblast cultures displayed the typical spindle-shaped morphology of proliferative endometrial fibroblasts. They expressed the appropriate lineage-specific markers (vimentin, CD90 and alpha-smooth muscle actin) but lack expression of epithelial markers (cytokeratin 8, E-cadherin and EpCAM) [26,30]. Further, the presence of mRNA for estrogen and progesterone receptors as well as the mRNA for two commonly secreted proteins (PAEP and MMP1) indicates that these fibroblasts reflect their *in vivo* phenotype throughout the 10 passages of culture on plastics [35,36]. While further investigation is warranted to determine their responsiveness to hormones, our observation suggests that these CAFS cells may provide an appropriate model to study the role of fibroblast in endometrial cancer progression.

Using CAFs from human endometrial cancer tissues, we showed that fibroblasts within the endometrial tumor microenvironment exhibit a pro-tumorigenic effect, by promoting the growth of endometrial cancer cell lines as well as primary endometrial cancer cell cultures. These effects are distinctly different to those isolated from non-tumor endometrial tissues. Several studies elegantly demonstrated that that stromal cells isolated from proliferative normal endometrium are capable of suppressing the growth of Ishikawa endometrial cancer cell line, even in response to estrogen and in cultures on basement membrane [26,27]. Such effects were specific to stromal cells derived from normal endometrium, since fibroblasts from normal foreskin failed to exhibit similar effects [26]. Likewise, the tumor-promoting effects we observed in CAFs are specific; fibroblasts obtained from endometrial hyperplasia tissue isolated using similar method did not demonstrate similar tumor-promoting effects.

Stromal reaction, especially expansion of fibroblasts, is not uncommon in tumor tissues. Recently, this phenotype has been correlated with advanced disease stage and poorer prognosis in many tumor types [37–39]. Fibroblasts from pancreatic tumors were shown to markedly contribute to tumor cell proliferation, motility, invasion and chemoresistance [12]. In an *in vivo* setting, CAFs from prostate tumors were capable of transforming genetically abnormal but non-tumorigenic benign prostate epithelial cells [11]. These fibroblasts are thought to secrete various cytokines and growth factors to activate proliferation and survival signaling pathways [40]. Furthermore, these cells may produce matrix metalloproteinases that could lead to extensive tissue remodeling that may cause increased angiogenesis and dysregulation of immune and inflammatory responses [22,23,41–43]. How the tumor microenvironment influences these fibroblasts to exhibit pro-tumorigenic properties, remain to be investigated. Studies from other cell models suggest that molecular changes can occur in these bystander cells to favor tumorigenesis [44,45].

Our data suggest that regulation of PI3K/Akt and MAPK/Erk survival pathways may be a key factor in the differential fibroblasts effects on endometrial cancer cell proliferation. We observed that these two pathways were inhibited when the endometrial cancer cells were exposed to secretion from normal endometrial fibroblasts (T-HESC). This is consistent with a recent study which demonstrated the suppression of PI3K/Akt but not MAPK/Erk in estrogen-stimulated Ishikawa cells, after treatment with supernatants from primary normal endometrial fibroblasts [27]. Interestingly, these two pathways were not suppressed, but activated by secretion from CAFs in our study. Using specific inhibitors to PI3K or MAPK, we further showed that CAFs-mediated tumor cell proliferation was in part, mediated by the activation of PI3K/Akt and MAPK/Erk.

Activation of PI3K pathway has been reported in up to 83% of EC cases, triggered by the loss of function of its key negative regulator, PTEN (phosphatase and tensin homolog deleted in chromosome ten) [46]. Consequently, several kinases including the serine/threonine kinase mTOR (mammalian target of rapamycin) became hyperactivated, leading to upregulation of anti-apoptotic proteins such as Bcl-2 [46]. In fact, dysregulation of this pathway has been implicated to confer resistance to conventional therapies [47]. There have been initiatives to use rapamycin in combination with hormonal and/or cytotoxic agents to improve treatment outcome [48,49]. Rapamycin has been shown to control transcription and translation process and thus affect cell cycle progression [50]. Our findings suggests that targeting CAFs may be a mode of action by which rapamycin (and its analogues i.e. ridaforolimus) in controlling endometrial cancer progression in the clinical setting [51,52].

Both PI3K and MAPK pathways have been associated with stimulation of external growth factors and cytokines [53,54], which can be found in both CAFs as well as normal fibroblasts. Comparison of the secretory factors expressed by CAFs and normal fibroblast revealed that MCP-1, RANTES, VEGF, IL-6 and IL-8 may individually or collectively activate these pathways to induce tumor cell proliferation. While MCP-1 and RANTES are shown to induce infiltration of immune cells and promote tumor invasion and metastasis [55–58], few evidence linked these two factors directly to tumor cell proliferation. Interestingly, activation of CCR5 by RANTES was thought to activate NFκB signaling via PI3K/Akt pathway to induce migration of human lung cancer and osteosarcoma cells [59,60]. Increased levels of VEGF have been associated with poorer outcome of women with endometrial cancer [61,62], and this cytokine may directly interact with PI3K pathway to promote lymphangiogenesis [63]. It is also worthy to note that increased VEGF level in CAFs secretion may induce EC cell proliferation, as shown recently by studies in breast cancer cells [64]. It remains to be investigated whether any of these cytokines are directly involved to induce EC cell proliferation.

Interleukin-6 and -8, both highly secreted by endometrial CAFs, promote the growth of various tumor types including colon, multiple myeloma and non-small cell lung cancers [65–69]. Although IL-8 was secreted quite equivalently by both CAFs and normal fibroblasts, studies showed that it can trigger PI3K and MAPK pathways to induce proliferation of endothelial and non-small cell lung cancer cells, respectively [70,71]. Similarly, inhibition of IL-6 pathway abrogated Stat3-mediated cell survival of gastric cancer and osteosarcoma [72,73], suggesting the importance of IL-6 in promoting tumor growth. Recently, phosphorylated-Stat3 expression in the tumor stroma, an indication of IL-6-JAK pathway activation, was thought to be a critical contributor to cancer progression and response to therapy by modulating PI3K pathway [74,75]. Nevertheless, few evidence are available to implicate the direct roles of these cytokines to EC cell proliferation.

It remains unknown, how secretion from different fibroblast population (normal versus cancer) can trigger explicit effects on the growth of endometrial cancer cells. It is evident that further investigation about the soluble factors identified in this study as well as other recently highlighted tumor fibroblasts secretory factors such as transforming growth factor (TGF) beta and stromal-derived factors (SDF)-1 [76,77], may provide some clues to these phenotypes. It is also important to understand the mechanisms by which the normal fibroblasts switch from tumor inhibitory to acquiring pro-tumor properties. It is possible that the stromal-epithelial interaction during carcinogenesis leads to the loss of ability to synthesize inhibitory factors [78]. Studies that compare the effects of CAFs and normal fibroblast may yield novel therapeutic targets for treating endometrial cancer.

## Conclusion

This study demonstrates that CAFs derived from endometrial cancer tissue are able to promote endometrial cancer cell proliferation, partly by activating PI3K and MAPK signaling pathways.

## Supporting Information

Figure S1 Effects of LY294002 and U0126 on ECC cell proliferation in the absence and presence of CAFs-conditioned media. ECC-1 (A) and EC6-Ep (B) cultured in control media containing 2% FBS were treated with either PI3K pathway selective inhibitor (LY294002), or Erk pathway selective inhibitor (U0126) for 72 hours. Similarly, additional EC cells (HEC-1A and EC14-Ep) were treated with LY294002 and U0126 in the absence (**C**, **D**) or presence of cancer-associated fibroblasts conditioned media (1 µg/µl) for 72 hours (**E**, **F**). Data shown are cell viability after normalized with control (media containing 2% FBS). Data, average; error bars, S.E.M. No significance observed between treated cells with control media for A-D. *, *P*<0.05; **, *P*<0.0001, when compared to CAFs-treated cells. Data shown are representative of three independent experiments.
(TIF)

Figure S2 Effect of rapamycin on CAFs-mediated cell proliferation in HEC-1A and EC14-Ep cell. HEC-1A cell line (**A**) and EC14-Ep primary epithelial cell (**B**) were treated with either control media (media containing 2% FBS) or 1 µg/µl EC11-Fib conditioned–media, in the presence of increasing dose of rapamycin for 72 hours. Data, average; error bars, S.E.M. *, *P*<0.0001 when compared to EC11-Fib treated cells. Data shown are representative of three independent experiments.
(TIF)

Figure S3 Measurement of individual cytokine for T-HESC and CAFs. Ten different cytokines were measured in the secretion of T-HESC and individual CAFs (EC6-Fib, EC7-Fib and EC11-Fib) in comparison with the control media (media containing 2% FBS), using an antibody array: (**A**) macrophage chemoattractant protein-1 (MCP-1); (**B**) interleukin (IL)-8; (**C**) matrix metalloproteinase-9 (MMP-9); (**D**) IL-10; (**E**) RANTES; (**F**) IL-12p70; (**G**) vascular endothelial growth factor (VEGF); (**H**) IL-13; (**I**) IL-6; and (**J**): interferon gamma (IFNg). Left graph showed the cytokines levels measured from each cell (pg/ml), while the right graph showed the standard curve graph (log(signal-background) *vs*. log(concentration)) for each cytokine. Data, average; error bars, S. EM. Data shown are average of fluorescence intensity from four array wells.
(TIF)
