# Supplementary figures and images for: Cancer-Associated Fibroblasts Promote Proliferation of Endometrial Cancer Cells
Source: PLoS One. 2013 Jul 26;8(7):e68923. doi: 10.1371/journal.pone.0068923 (PMC3724864; doi:10.1371/journal.pone.0068923)

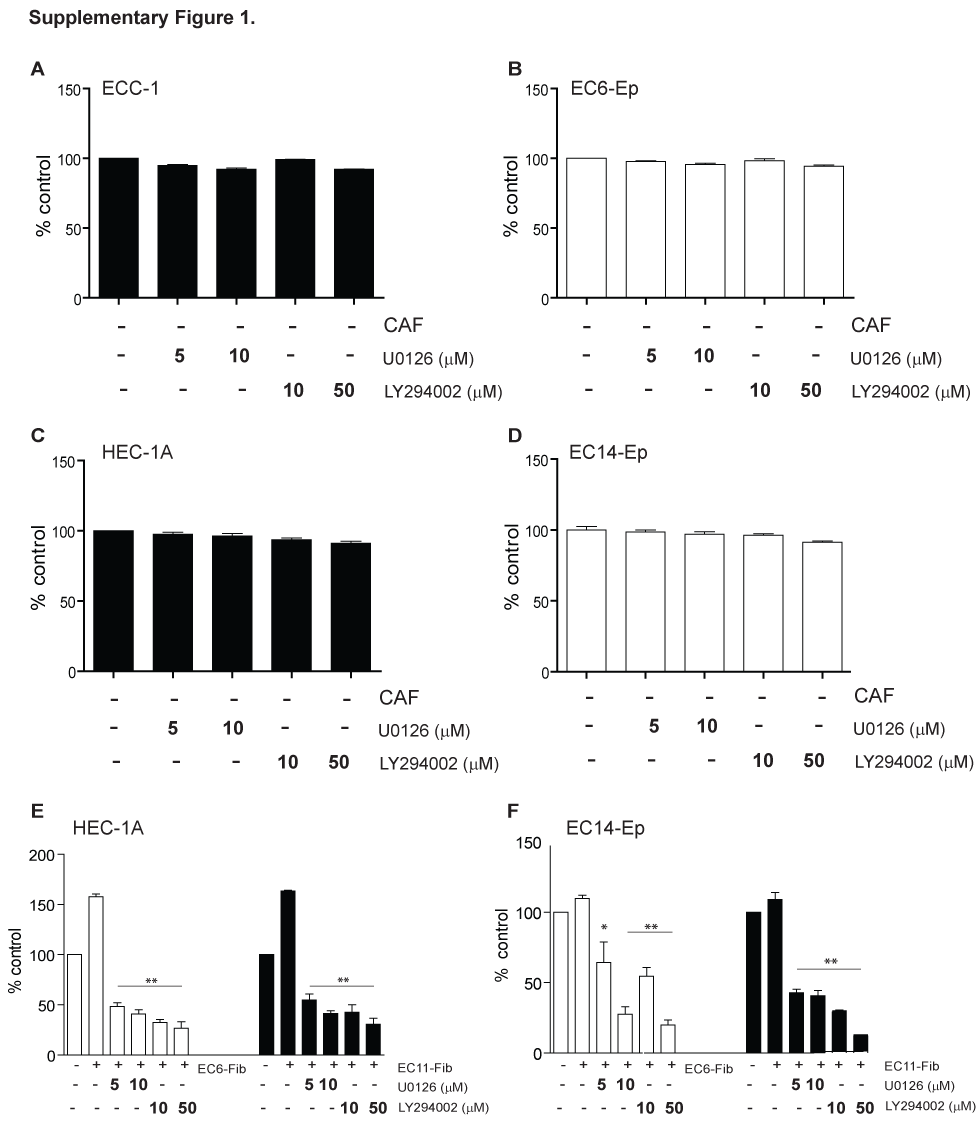

Supplement: Figure S1 — ECC-1 (A) and EC6-Ep (B) cultured in control media containing 2% FBS were treated with either PI3K pathway selective inhibitor (LY294002), or Erk pathway selective inhibitor (U0126) for 72 hours. Similarly, additional EC cells (HEC-1A and EC14-Ep) were treated with LY294002 and U0126 in the absence (C, D) or presence of cancer-associated fibroblasts conditioned media (1 µg/µl) for 72 hours (E, F). Data shown are cell viability after normalized with control (media containing 2% FBS). Data, average; error bars, S.E.M. No significance observed between treated cells with control media for A-D. *, P<0.05; **, P<0.0001, when compared to CAFs-treated cells. Data shown are representative of three independent experiments. (TIF) [file pone.0068923.s001.tif]

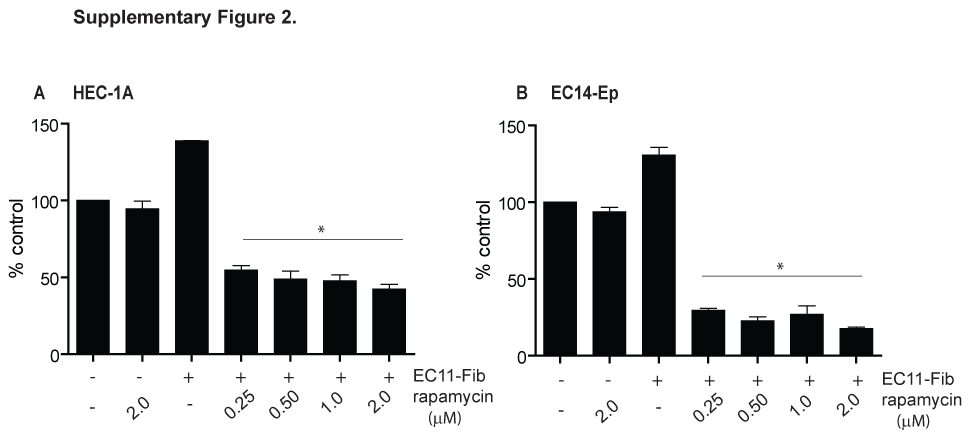

Supplement: Figure S2 — HEC-1A cell line (A) and EC14-Ep primary epithelial cell (B) were treated with either control media (media containing 2% FBS) or 1 µg/µl EC11-Fib conditioned–media, in the presence of increasing dose of rapamycin for 72 hours. Data, average; error bars, S.E.M. *, P<0.0001 when compared to EC11-Fib treated cells. Data shown are representative of three independent experiments. (TIF) [file pone.0068923.s002.tif]

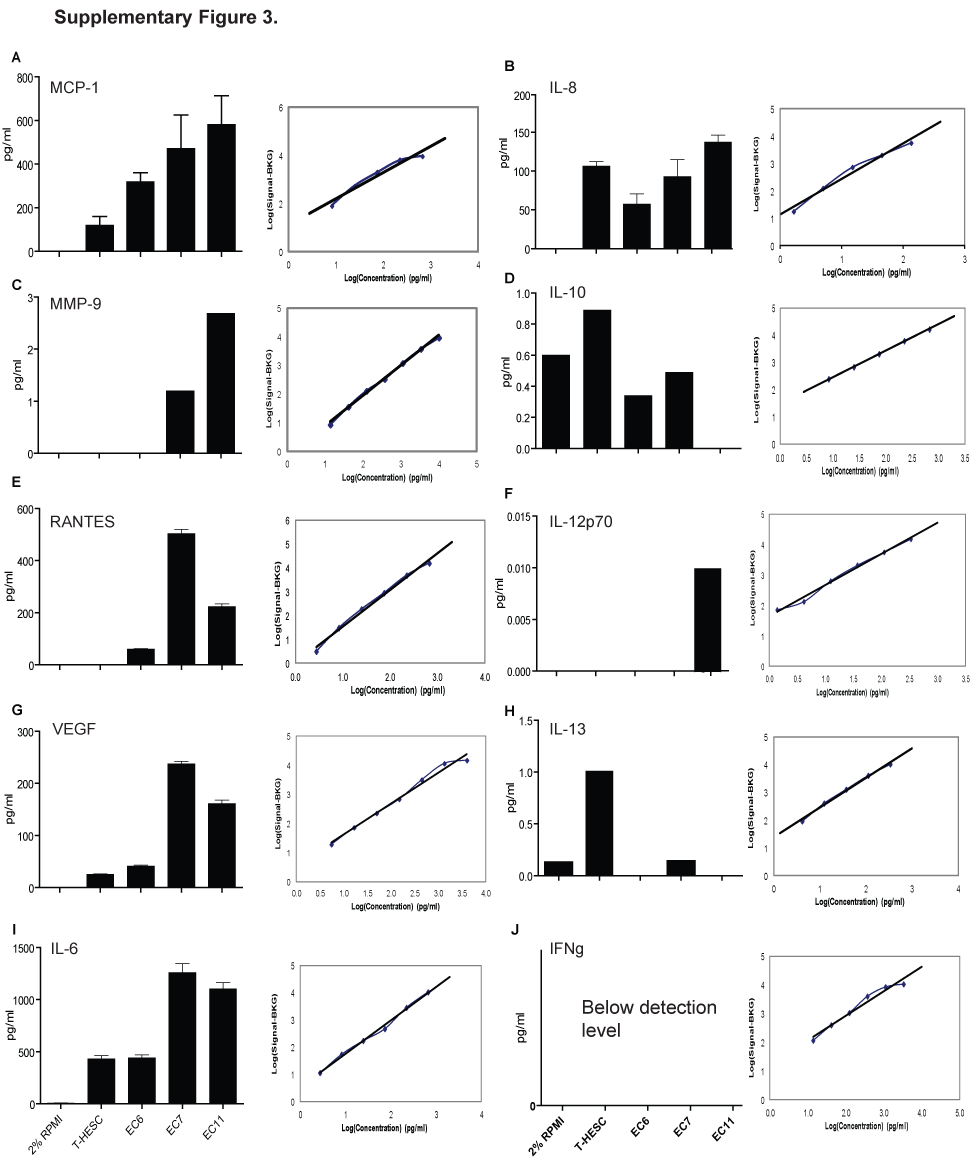

Supplement: Figure S3 — Ten different cytokines were measured in the secretion of T-HESC and individual CAFs (EC6-Fib, EC7-Fib and EC11-Fib) in comparison with the control media (media containing 2% FBS), using an antibody array: (A) macrophage chemoattractant protein-1 (MCP-1); (B) interleukin (IL)-8; (C) matrix metalloproteinase-9 (MMP-9); (D) IL-10; (E) RANTES; (F) IL-12p70; (G) vascular endothelial growth factor (VEGF); (H) IL-13; (I) IL-6; and (J): interferon gamma (IFNg). Left graph showed the cytokines levels measured from each cell (pg/ml), while the right graph showed the standard curve graph (log(signal-background) vs. log(concentration)) for each cytokine. Data, average; error bars, S. EM. Data shown are average of fluorescence intensity from four array wells. (TIF) [file pone.0068923.s003.tif]
